# Allergic Contact Dermatitis in Children Over a 20‐Year Period: A Danish Single‐Centre Study

**DOI:** 10.1111/cod.70241

**Published:** 2026-09-16

**Authors:** Laura Krogh Herlin, Anne Bregnhøj, Mette Sommerlund

**Affiliations:** ^1^ Department of Dermatology Aarhus University Hospital Aarhus Denmark

**Keywords:** acrylates, allergic contact dermatitis, children, contact allergy, patch testing

Allergic contact dermatitis (ACD) is a common skin disease caused by delayed‐type hypersensitivity to environmental allergens [1]. While previously considered less prevalent in children, ACD is increasingly recognized as an important cause of chronic dermatitis in the paediatric population [2]. Despite growing awareness, long‐term longitudinal data on temporal trends in causative allergens remain scarce.

We examined the prevalence and pattern of contact allergy in a paediatric population over a 20‐year period.

## 1 Methods and Results

We conducted a retrospective study of all patients aged 0–18 years referred for patch testing at the Department of Dermatology, Aarhus University Hospital, Denmark, between January 2005 and December 2024. Patients were patch tested with the European baseline series (entire period) and/or a paediatric baseline series (since 2014, children from 0 to 12 years). Additional allergens, including acrylate allergens (since 2016), were applied based on clinical history and suspected exposures. The clinical relevance of positive patch test reactions was assessed by a trained dermatologist. Patch test outcomes were analysed overall and stratified across four consecutive 5‐year periods.

A total of 721 patients were included (median age 10.8 years; 41.8% male). Atopic dermatitis was present in 55.3%. A positive patch test was observed in 34.4% of patients, with stable prevalence over time: 37% (2005–2009), 36% (2010–2014), 29% (2015–2019), and 37% (2020–2024).

Metal allergens predominated, including nickel sulphate (13.0%), cobalt dichloride (8.7%), and potassium dichromate (7.8%). Other frequent allergens were methylisothiazolinone (3.6%) and fragrance mixes (Table 1). Clinical relevance was identified in 49.6% of positive patch test reactions.

**TABLE 1:** Common contact allergens among 721 tested patients from 2005 to 2024.

Allergen	Entire period		2005–2009		2010–2014		2015–2019		2020–2024	
	n/N	%	n/N	%	n/N	%	n/N	%	n/N	%
Any positive test	248/721	34.4	43/116	37.1	61/170	35.9	63/218	28.9	81/217	37.3
Nickel sulphate	94/721	13.0	18/116	15.5	28/170	16.5	19/218	8.7	29/217	13.4
Cobalt dichloride	63/721	8.7	6/116	5.2	11/170	6.5	21/218	9.6	25/217	11.5
Potassium dichromate	56/721	7.8	2/116	1.7	12/170	7.1	17/218	7.8	25/217	11.5
Methylisothiazolinone	26/721	3.6	4/116	3.5	7/170	4.1	9/218	4.1	6/217	2.8
Sesquiterpene lactone mix	24/721	3.3	6/116	5.2	6/170	3.5	7/218	3.2	5/217	2.3
Fragrance mix I	21/721	2.9	4/116	3.5	5/170	2.9	6/218	2.8	6/217	2.8
Fragrance mix II	15/721	2.1	3/116	2.6	4/170	2.4	6/218	2.8	2/217	0.9

Testing with acrylates increased in recent years, especially from 2020. Positive reactions were present in 12 of 101 (11.9%) patients tested.

## 2 2Discussion

The stable prevalence of positive patch tests across the study period (30%–40%) is consistent with findings from other European centres suggesting, that the overall burden of contact sensitization in children has not changed significantly over two decades [3].

Metal allergens continue to dominate, suggesting persistent exposure despite preventive measures [4]. The emergence of acrylate allergy may reflect changing exposures among children and adolescents, including artificial nails and adhesives used in continuous glucose monitoring sensors [5, 6].

These findings support continued patch testing in children and highlight the need to adapt allergen panels to evolving exposures in this population. Increased awareness of acrylate allergy is warranted. We welcome the proposed European paediatric baseline series to standardize patch testing in children [7].

## Author Contributions

**Laura Krogh Herlin:** conceptualization, writing – original draft, formal analysis, investigation. **Anne Bregnhøj:** writing – review and editing, supervision, data curation. **Mette Sommerlund:** supervision, writing – review and editing, data curation, conceptualization.

## Funding

The authors have nothing to report.

## Ethics Statement

The study was based on data from a continuously updated clinical quality database at the Department of Dermatology, Aarhus University Hospital, which prospectively registers all patients undergoing patch testing. In accordance with Danish legislation, register‐based studies using data from clinical quality databases do not require approval from an ethics committee (Committee Act §14, stk. 2). Data were handled and stored in accordance with the General Data Protection Regulation.

## Conflicts of Interest

The authors declare no conflicts of interest.

## Data Availability

The data that support the findings of this study are available from the corresponding author upon reasonable request.
